# Dependence of track etching kinetics on chemical reactivity around the ion path

**DOI:** 10.1038/s41598-019-51748-y

**Published:** 2019-10-25

**Authors:** S. A. Gorbunov, R. A. Rymzhanov, A. E. Volkov

**Affiliations:** 1 P. N. Lebedev Physical Institute of the Russian Academy of Sciences, Leninskij pr. 53, 119991 Moscow, Russia; 2 Joint Institute for Nuclear Research, Joliot-Curie 6, 141980 Dubna, Moscow Region Russia; 3 The Institute of Nuclear Physics, Ibragimov St. 1, 050032 Almaty, Kazakhstan; 4 National Research Centre ‘Kurchatov Institute’, Kurchatov Sq. 1, 123182 Moscow, Russia; 5 National University of Science and Technology MISiS, Leninskij pr., 4, 119049 Moscow, Russia

**Keywords:** Astrophysical dust, Particle astrophysics, Computational science, Techniques and instrumentation, Surface patterning

## Abstract

Etching kinetics of swift heavy ions (SHI) tracks in olivine is investigated in frame of experimentally verified numerical approach. The model takes into account variation of induced chemical reactivity of the material around the whole ion trajectory with the nanometric accuracy. This enables a quantitative description of wet chemical etching of SHI tracks of different lengths and orientations towards to the sample surface. It is demonstrated that two different modes of etching, governed by diffusion of etchant molecules and by their reaction with the material must be observed in experiments using techniques with different resolution thresholds. Applicability limits of the optical microscopy for detection of heavy ion parameters by measuring of the lengthwise etching rates of the ion track are discussed.

## Introduction

Being decelerated in solids, swift heavy ions (SHI) with masses *M* > 10 a.m.u., and energies *E* > 1 MeV/a.m.u. considerably excite the electron subsystem of a target (>95% of the total ion energy loss, *dE*/*dx* = *S*_*e*_ = 5–50 keV/nm^1–3^) along their trajectories. Relaxation of this excitation results in structure transformations in the nanometric vicinity of the ion path^1–8^. Changes in electronic and chemical properties of a target can appear at distances up to hundreds of nanometers from the track axis^9–12^. Utilizing effects of chemical activation, methods based on wet chemical etching (WCE) of SHI tracks were developed for fabrication of membranes, microdiaphragms, conductive channels, polymer filters, nanostructures and nanowires^8^, as well as for design of heavy particles detectors and analysis of results of their irradiations^12–18^.

In particular, WCE of ion tracks^12^ in crystalline olivine (Mg_0.11_Fe_0.89_)_2_SiO_4_ from meteorites pallasites is used to register parameters of heavy nuclei from galactic cosmic rays (GCR)^12,14,16,19^. After SHI passage, a track core of 1 ÷ 10 nm diameter^20^ containing highly damaged material and high concentrations of Fe^+^ cations reduced by spreading electrons appears along the heavy ion path in olivine. Ensemble of reduced Fe^+^ cations propagates up to distances of 100–300 nm from the ion trajectory forming a halo of the track core^21^. Reaction rates of etchant molecules with olivine in the track core are of orders of magnitude larger than those in outer halo regions. Competition and coupling of these two modes of chemical activation result in formation of a complex spatial distribution of chemical reactivity of the material around the SHI trajectories in olivine.

Track etching realizes as a cumulative effect of chemical reactions governed by the chemical reactivity at the interface of an appearing etched channel and the diffusion rates of molecules of an etchant along this channel. This leads to coexistence of two mechanisms of WCE of SHI tracks in (Mg_0.11_Fe_0.89_)_2_SiO_4_^21^: (a) fast etching of the structure transformed track core controlled by diffusion of etchant molecules to the etching front, and (b) slow etching of the track periphery containing Fe^+^ cations controlled by reaction rates of these molecules. Competition between these mechanisms occurs in the intermediate region. Relation between diameters of the etched track core and the periphery region depends on the spatial distribution of chemical reactivity generated by the deposited energy. It varies with changes of the ion velocity along the projectile path.

Models of WCE in minerals, formulated in 1960s^15^ and systematized in 1970s^17^, link linearly the track etching rate with the SHI energy losses^15^ along the ion trajectory^22–24^. On the other hand, recent experiments measuring the track etching parameters with nanometric resolution contradicted with these models. In particular, nonlinear dependence of lengthwise track etching rates on the ion energy losses was observed^25^.

To account for this effect, we developed a method^21^ coupling a microscopic model of excitation and relaxation of materials in SHI tracks^26^ with the chemical transition state theory and a WCE model. This approach describes: (1) excitation and relaxation kinetics of the electronic and ionic subsystems of a target in an SHI track^26–28^; (2) changes of chemical states of a material stimulated by this kinetics^13^ providing with the radial distributions of chemical reactivity around the SHI trajectories; and (3) a model of wet chemical etching of an SHI track taking into account diffusion-controlled WCE of the nanometric track core, reaction-controlled WCE at the larger distances from the ion trajectory, as well as gradual transition between these etching modes in the intermediate region. But this approach^21^ could not describe effects of a lengthwise heterogeneity of the radial distribution of chemical reactivity of a material along the SHI trajectory. As a result, application of this WCE model^21^ gave only estimations of the lengthwise etching rate (LER) of an SHI track which appeared very similar to those detected in irradiated olivine crystals^12,21^.

This stimulates us to improve the developed model^21^ to be able to simulate WCE of materials along the whole SHI path. Applying this new ability we present in this paper time resolved modeling of the etching kinetics of Xe and U ions tracks of different lengths and directions in olivine. It is also demonstrated that observed LER depends crucially on the spatial resolution of methods used for etched track detection. In particular, application limits of the optical techniques for registration of ions by measuring LERs of fast ion tracks in olivine^15,17,22,29,30^ are investigated.

## Modeling of Chemical Etching of SHI Tracks

**Figure 1:**
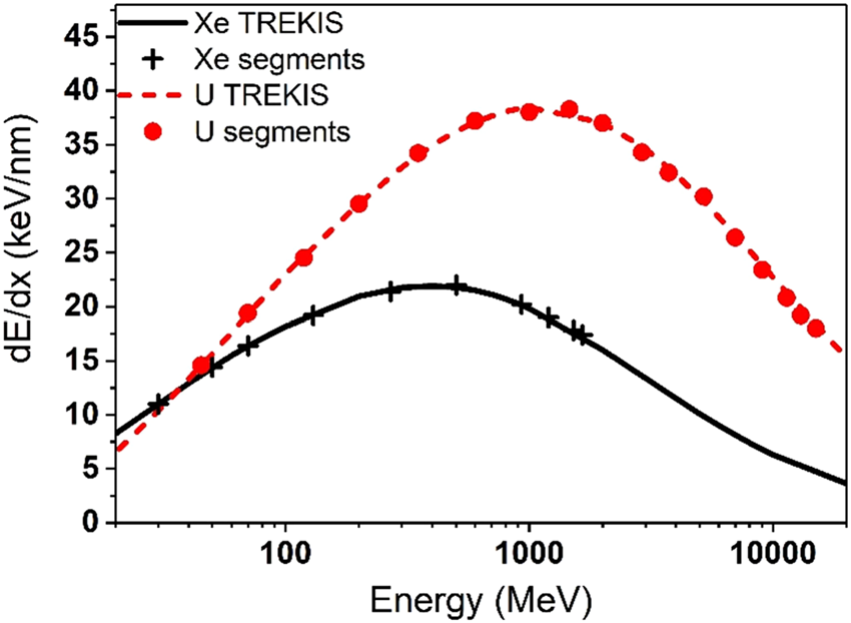
Energy losses of U and Xe ions of different energies in olivine. *dE/dx* calculated with TREKIS code^27^.

In the presented approach the whole ion path is divided into segments corresponding to changes of the electronic energy losses (d*E*/d*x)* of the ion. These energy values for Xe and U ions in olivine are listed in Table 1 and shown in Fig. 1.

**Table 1:** Energies and energy losses of 16 GeV (67.5 MeV/a.m.u) U and 1.7 GeV (12.9 MeV/a.m.u) Xe ions in olivine as functions of the residual range *RR* (see Fig. 2 and explanation below).

U ion (16 GeV)			Xe ion (1.7 GeV)		
Residual range (*RR*), μm	Ion energy, MeV/a.m.u	*dE/dx*_*e*_, keV/nm	Residual range (*RR*), μm	Ion energy, MeV/a.m.u	*dE/dx*_*e*_, keV/nm
4.93	0.19	14.6	4.74	0.23	11
6.34	0.29	19.4	6.22	0.38	14.4
8.35	0.5	24.5	7.47	0.53	16.4
11.1	0.84	29.5	10.61	0.99	19.2
15.57	1.47	34.2	17.17	2.06	21.4
22.45	2.52	37.2	27.78	3.83	22
32.95	4.20	38.0	48.38	7.10	20.2
45.4	6.13	38.3	64.39	9.16	19.0
59.95	8.40	37.0	84.24	11.59	17.8
85.96	12.18	34.3	92.48	12.60	17.4
111.7	15.70	32.4			
160.18	21.85	30.2			
226.11	29.41	26.4			
309.59	38	23.4			
443.33	47.90	20.8			
548.02	54.62	19.2			
678.88	63.03	18.0			
750	67.5	17.2

**Figure 2:**
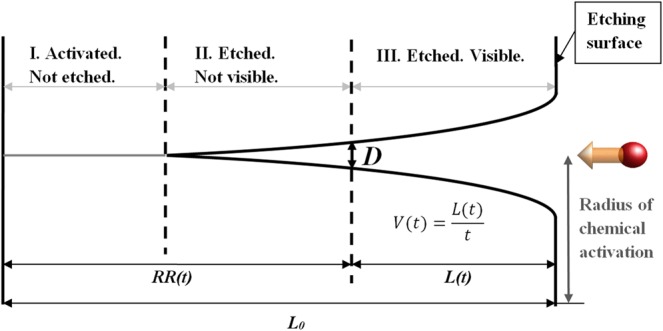
Dependence of the lengthwise etching rate (*LER*) on the residual range (*RR*) of an ion. *L(t)* is the length of the visible part of an etched track, while *L*_0_ is the total ion range. *t* is the etching time. *D* is the visibility threshold of detection of an etched channel by an applied technique.

We define^12^ the lengthwise etching rate (LER) as the detected etched length *L(t)* divided by the etching time *t* (Fig. 2). Figure 2 also illustrates the definition of the residual range (*RR*) as a distance from the observed part *L(t)* of etched track to the stopping point of an ion. The *RR* is a commonly used parameter when analyzing results of etching of olivine from pallasites, because the stopping point of track etching is often well detected in experiments^12^.

**Figure 3:**
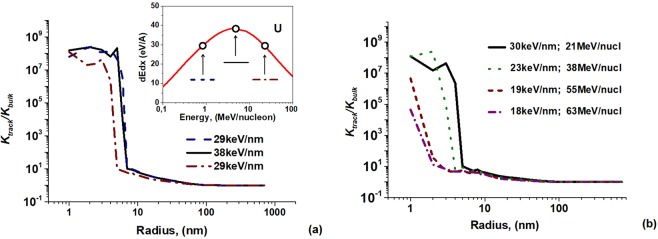
Relative reaction rates of olivine with an etchant in the vicinity of the trajectories of U ions of different energies. *K*_*track*_ is the reaction rate in a chemically modified track, *K*_*bulk*_ is that for the undamaged olivine.

The model developed in^21^ is applied to calculate the radial dependence of induced chemical reactivity of olivine in these segments of the SHI trajectory. This model couples the original Monte-Carlo TREKIS code^27^ describing excitation of the electronic system of a material, its interaction with the lattice and molecular dynamics modeling  (LAMMPS code^31^) of subsequent relaxation of the excited atomic subsystem with the transition state theory^32,33^. High level of material excitation results in considerable structure transformations and formation of large concentrations of Fe^+^ cations reduced by flying apart electrons, which cause considerable changes of chemical reactivity in the track core at distances up to 1–5 nm from the ion trajectory. Elastic fields from this core and created Fe^+^ cations form a halo of lowered barriers of chemical reactions of etchant molecules with an excited olivine at distances up to ~100–300 nm^13,21^ from the track axis. Figure 3 presents an example of such calculations for U ions of different velocities in olivine.

**Figure 4:**
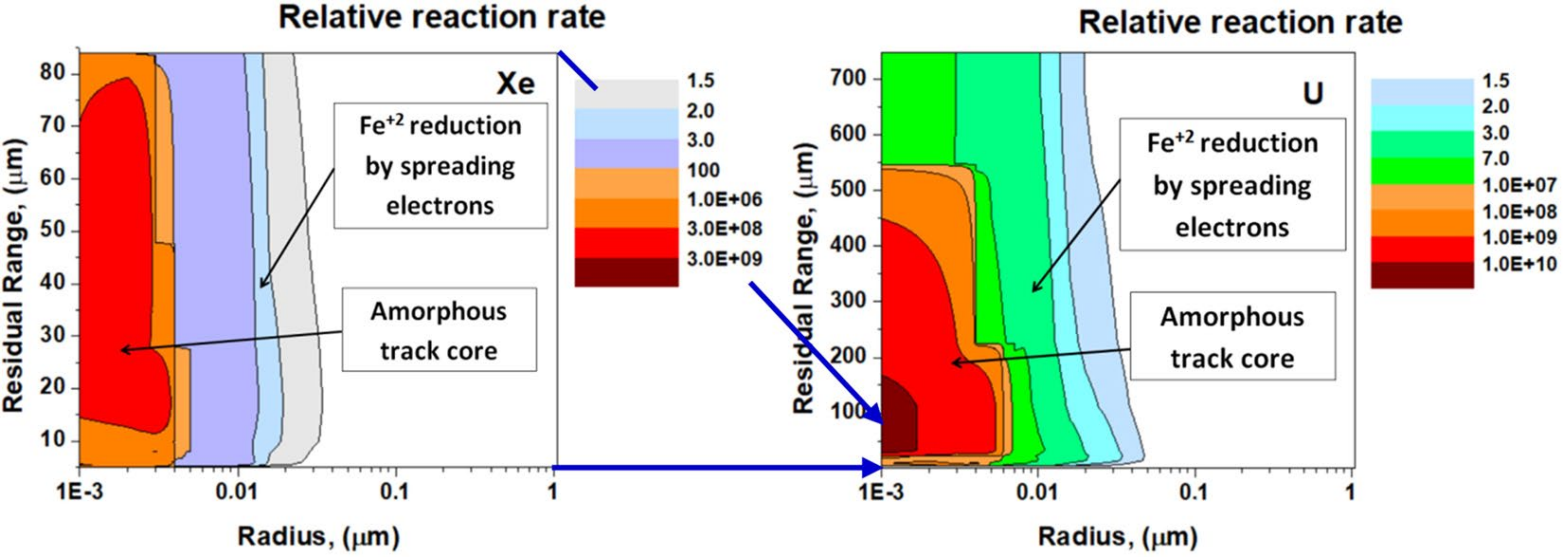
Distributions of the relative reaction rates of olivine with an etchant in the vicinities of the trajectories of 1.7 GeV Xe and 16 GeV U ions. Blue arrows between the left and right panels indicate the difference between the lengths of these tracks.

Then, bilinear interpolation^34^ is applied to link together the calculated radial distributions of chemical reactivity in the ion trajectory segments and describe activation of olivine in intermediate points. This modeling step allows obtaining 3D distribution of chemical reactivity of excited olivine along the ion path. Resulting distributions of chemical reactivity around the trajectories of uranium and xenon ions with the initial energies of 16 GeV and 1.7 GeV respectively are presented in Fig. 4. These energies correspond to the threshold energy loss (~16 keV/nm) necessary to produce structure transformations of olivine (see details in ref.^20^). The ranges of these ions (750 μm for U and 85 μm for Xe) were used as the total track lengths for the modelling of etching.

Figure 4 illustrates that the radii of highly chemically activated regions induced by the applied ions differ only by 2 times, whereas the lengths of the chemically activated tracks of these ions differ by 10 times.

**Figure 5:**
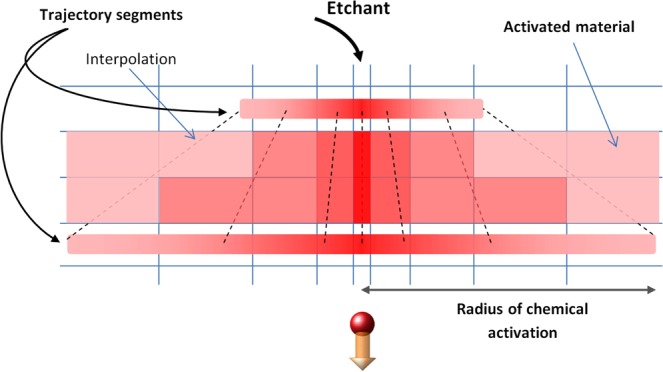
Schematic projection of the 3D chemical reactivity distribution on the spatially irregular numerical grid for WCE modeling.

Finally, the obtained distribution of chemical reactivity is projected to a spatial numerical grid, which is irregular in the both directions: along the projectile path (Table 1 and Fig. 1) and the radial one (see Fig. 5). This allows effective and fast simulating of WCE along the whole SHI path taking into account the generated spatial distribution of chemical reactivity of the material in an ion track.

**Figure 6:**
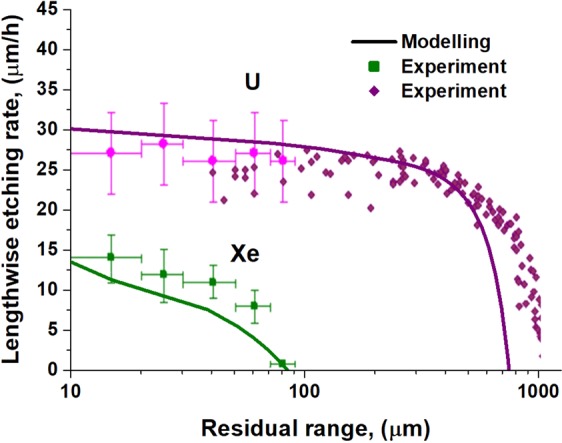
Lengthwise etching rates as functions of the residual ranges of 1.7 GeV Xe and 16 GeV U ions in (Mg_0.11_Fe_0.89_)_2_SiO_4_. Solid lines present the LERs obtained from the modeling. Experimental values are indicated by squares and rhombuses with error bars^14^. Registration threshold of 400 nm is assumed.

To verify the renewed model, we compared the calculated LERs along the paths of Xe and U ions with the OLYMPIA-experiment calibration database^12,14^ for olivine crystals from meteorites. Figure 6 demonstrates a good agreement between the calculated LERs with the experimental results when the optical microscopy visibility threshold of 400 nm is assumed. It should be noted that no fittings were applied when calculating these LERs.

## Applicability of the Optical Technique for Registration of Etched Tracks

Optical technique is usually used for determination of parameters of etched tracks of heavy nuclei from GCR. Different orientations of these tracks are observed in etched meteorite detector^12,14,16,19^. Due to spatial inhomogeneity of the induced chemical reactivity along SHI trajectories different etching kinetics may realize for tracks differently oriented relative to the sample surface.

Moreover, registration of tracks of identical heavy nuclei having different energies is a routine case for the WCE method of cosmic rays detection in small (1–2 mm) olivine crystals contained in meteorites^12^. Indeed, passage of a projectile through surrounding crystals and metallic matrix reduces the ion velocity. Therefore, not only various orientations of the ion trajectories, but also different initial projectile energies resulting in different track lengths must be taken into account when identifying ion parameters from the etching data of a selected crystal. To study the possibility of the optical method to resolve such differences, we investigated an effect of different orientations and lengths of U and Xe ion tracks.

**Figure 7:**
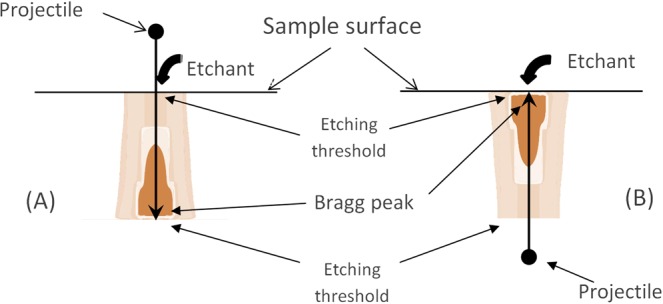
Orientations of tracks of swift heavy ions: (**A**) the ion entrance point locates at the initial interface of a crystal with an etchant, and (**B**) the Bragg peak of the energy losses of an ion is close to this interface.

**Figure 8:**
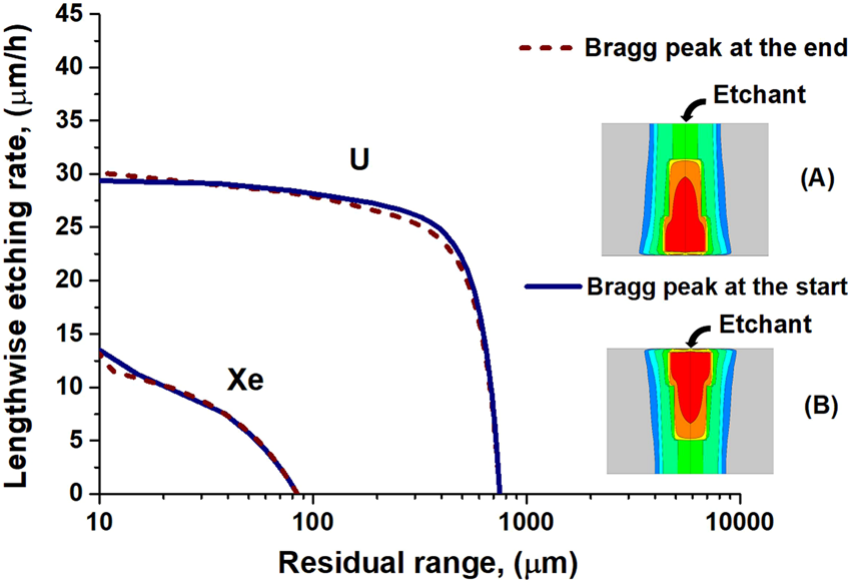
Lengthwise etching rates of U and Xe tracks in olivine as the functions of the residual range depending on the position of the Bragg peak relative to the initial interface of a crystal with an etchant. 400 nm is assumed as the visibility threshold of the etched channel.

Figures 7 and 8 demonstrate only a little difference between calculated LER dependencies for opposite directed tracks of these ions on the residual range when 400 nm is assumed as the visibility threshold of the etched channel. This suggests the applicability of the optical microscopy for the identification of GCR nuclei by oppositely directed etched tracks in olivine.

**Figure 9:**
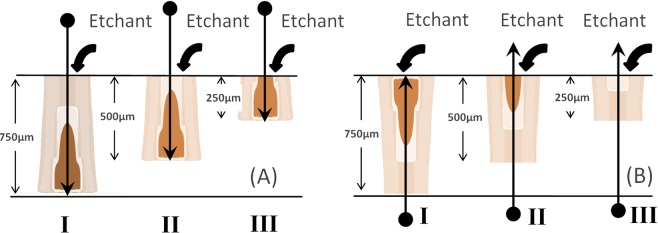
Etching of U tracks of different lengths in olivine detectors. (**A**) The entrance point of an ion into a crystal is at the surface from which etching begins, and (**B**) the end of the track is close to this surface.

**Figure 10:**
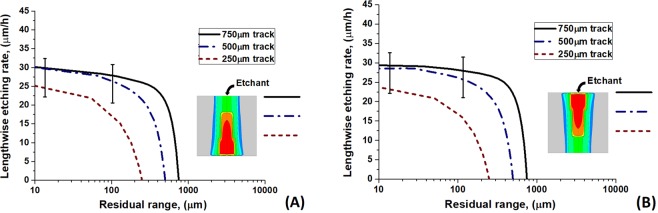
Lengthwise etching rates of U ion tracks of different lengths (750 μm, 500 μm and 250 μm) in olivine as functions of the residual ranges. Vertical lines present the experimental errors (see Fig. 6). Effect of different orientation of these tracks relative to the etching surface is illustrated by the left and right panels. 400 nm is assumed as the visibility threshold of the etched channel.

Modeling of etching of tracks of U ions of 750 μm (initial ion energy is 67.5 MeV/amu), 500 μm (50 MeV/amu) and 250 μm (31 MeV/amu) total lengths (Fig. 9) demonstrates (Fig. 10) that the optical microscopy can supply with a reliable information about the ion parameters only for LER at *RR* < 50–100 μm even for long tracks. These results also demonstrate that 250 μm can be treated as the threshold length of a track necessary for extraction of information about projectile parameters by the WCE technique when the visibility threshold ~400 nm is used. For such short tracks LER at *RR* < 20 μm should be analyzed to extract the ion parameters.

**Figure 11:**
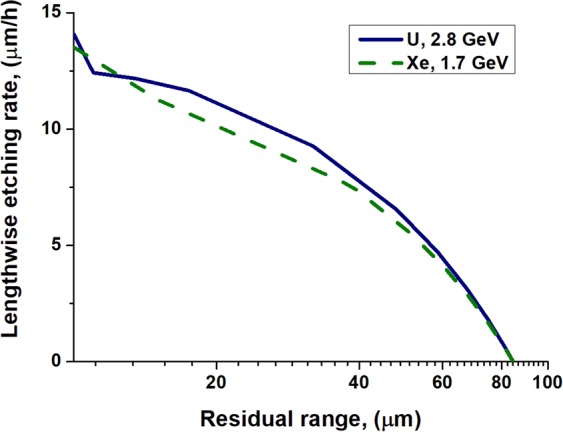
Lengthwise etching rates of 2.8 GeV U and 1.7 GeV Xe tracks having the same lengths of 85 μm as the functions of the residual ranges. 400 nm is assumed as the visibility threshold of the etched channel.

To illustrate the latest point we also performed simulations of etching of latent tracks of 1.7 GeV Xe and 2.8 GeV U ions. Having twice as different the initial electronic energy losses (see Table 1), these ions produce etched tracks of the same length (85 μm) in olivine. Figure 11. demonstrates only a minor difference between the calculated LER dependencies on *RR* for tracks of these ions when the optical microscopy is used for etched channels registration. This indicates that the optical technique cannot identify ions producing such short tracks in olivine.

**Figure 12:**
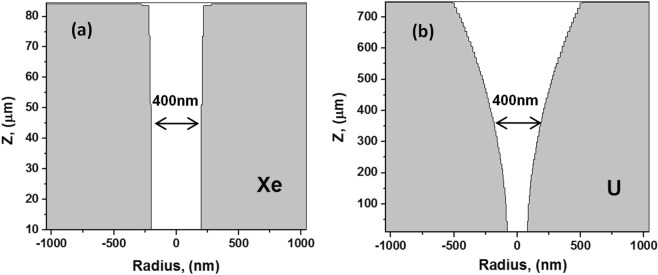
Shapes of 1.7 GeV Xe and 16 GeV U etched tracks by the time when the detected length of an etched track is equal to a half of the total length of a channel which can be etched.

To analyze the problem of application of the optical microscopy we present Fig. 12 demonstrating a difference between shapes of long 16 GeV U and short 1.7 GeV Xe tracks by the time when the detected length of an etched channel is equal to a half of the total length of a track.

- Short tracks
     Short etched tracks (1.7 GeV Xe ion, Fig. 12) appear as almost cylindrically shaped. This occurs due to fast etching of highly chemically modified olivine in the track core (see Fig. 4). This process is governed by diffusion of etchant molecules along the etched channel. After formation of a narrow nanometric channel, further etching of such track occurs in the radial direction and is governed by an almost constant concentration of etchant molecules along the track axis. Etching of a track core halo (r > 20 nm) is controlled by the reaction rate of pure or slightly excited olivine with an etchant. Such slow etching of the periphery needs much larger time to reach the diameter of 400 nm than to complete etching of the core of short tracks and depends weakly on projectile parameters.
  As a result, regardless the difference between the diameters of the initially etched cores the optical microscopy cannot detect a difference between the lengthwise etching rates of short tracks of Xe and U ions (see Fig. 11).
- Long tracks

   For longer tracks (U ion, the right panel in Fig. 12), the etching front needs a significant time to reach the track core at deeper parts of the ion path (>300–400 μm) because of a finite diffusion rate of etchant molecules. This results in much longer time available for the radial etching near the crystal surface causing a cone-like shape of the etched channel. In addition, the concentration of etchant in vicinity of the crystal surface is always higher than that in the deeper parts of a long track because of consumption of etchant molecules by their reaction with the walls of an etched channel. This decelerates track core etching and enhances the conicity of the track shape, which determined by the projectile parameters.

## Dependence of the Track Etching Parameters on the Visibility Threshold

Simulations presented in the previous Section demonstrated disadvantages of application of the optical microscopy for investigations of effects of radial inhomogeneity of the distribution of chemical reactivity along the projectile trajectory on the etching kinetics of SHI tracks. Indeed, drastic changes of the etching rates around the trajectories of different ions appear only inside the region of the diameter of ~10 nm (see Fig. 4 and^20^). This diameter is much less than the optical resolution threshold limited by the wavelength of visible light (e.g. ~400 nm). Information about details of the etching kinetics of these highly chemically modified nanometric regions is crucially distorted by fast diffusion controlled etching, long before detection of etched channels by the optical microscopy.

**Figure 13:**
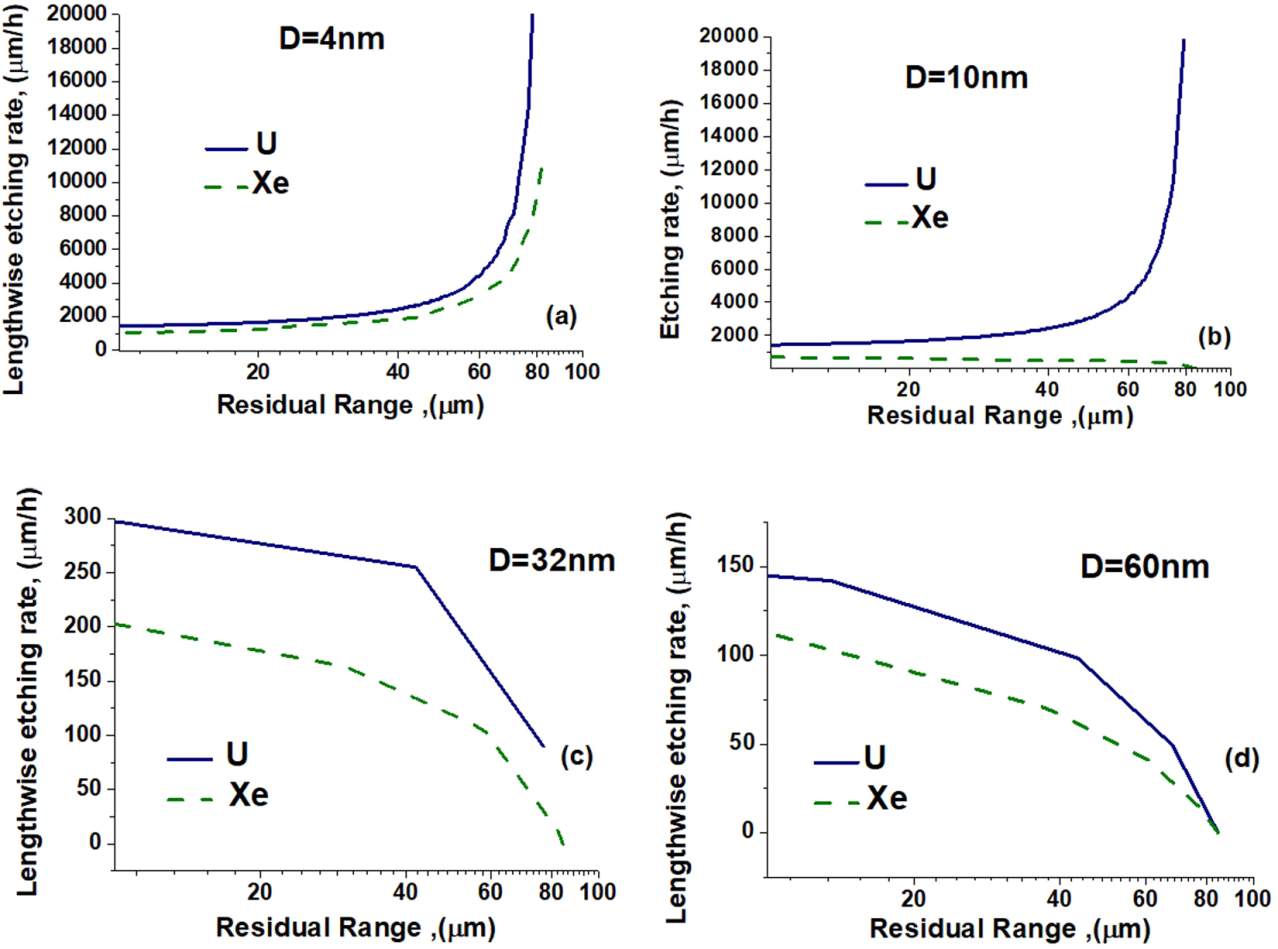
Lengthwise etching rates of U and Xe ion tracks of the same length (85 μm) in olivine as functions of the residual ranges. The visibility thresholds *D* are (**a**) 4, (**b**) 10, (**c**) 32 and (**d**) 60 nm.

Experimental techniques providing nanometric precision of etched channels detection can be applied for detailed monitoring of track etching kinetics, e.g. recently developed *in-situ* method^10^ of X-ray diagnostics without destroying samples. To demonstrate such a possibility, we simulated LERs of U and Xe tracks of the same length (85 μm) using the visibility thresholds ranging from 4 nm to 60 nm (Fig. 13).

The threshold *D* = 4 nm is smaller than the diameters of the highly chemically excited cores of tracks of the both ions. In this case, LER decreases when the residual range decreases, i.e. with an increase of the etched length (Fig. 13a). This occurs because the diffusion of etchant molecules deep into the channel needs longer times.

*D* = 10 nm is larger than the diameter of cylinder containing highly chemically activated olivine in the xenon track, but it is comparable with the size of this region in the uranium track. In this case two different modes of track etching are observed for Xe vs. U ions: (a) reaction controlled etching of the halo containing ionized Fe^+^ atoms in Xe tracks, and (b) much faster diffusion controlled etching of the track core in U tracks. Figure 13b illustrates this difference.

Reaction controlled etching of the halos of ionized Fe^+^ cations dominates in channels with diameters larger than the visibility threshold *D* = 32 nm for the both ion tracks (Fig. 13c). The observed LERs are much smaller than the rates of etching controlled by diffusion presented in Fig. 13a and in Fig. 13b for uranium. The concentration of Fe^+^ cations in U tracks is larger than that in Xe tracks, resulting in the larger detected LER values for U tracks.

Increase of the visibility threshold up to *D* = 60 nm (Fig. 13d) brings to the detection of the far halo regions of the both tracks. In these regions the concentrations of Fe^+^ cations are small resulting in low LERs. The difference between Fe^+^ concentrations in U and Xe tracks decreases causing reduction of the difference between the observed LER. Such tendencies are kept with subsequent increase of the visibility threshold. As it was demonstrated in the previous Section, at *D* = 400 nm almost no difference between LER is observed for tracks of U and Xe ions of 85 μm lengths (see Fig. 11).

Summarizing Fig. 13, two kinds of the LER(*RR*) dependences can be observed for different visibility thresholds of the etched tracks. When the minimum observable diameter of the etched channel is smaller than the size of the area of diffusion-controlled etching, the LER decreases when RR decreases. This occurs because of the decrease of the diffusion rate of etchant molecules with the rise of the length of an etched channel. In the opposite case LER increases when *RR* decreases because the etched track becomes visible much later than etching of highly activated region finishes.

**Figure 14:**
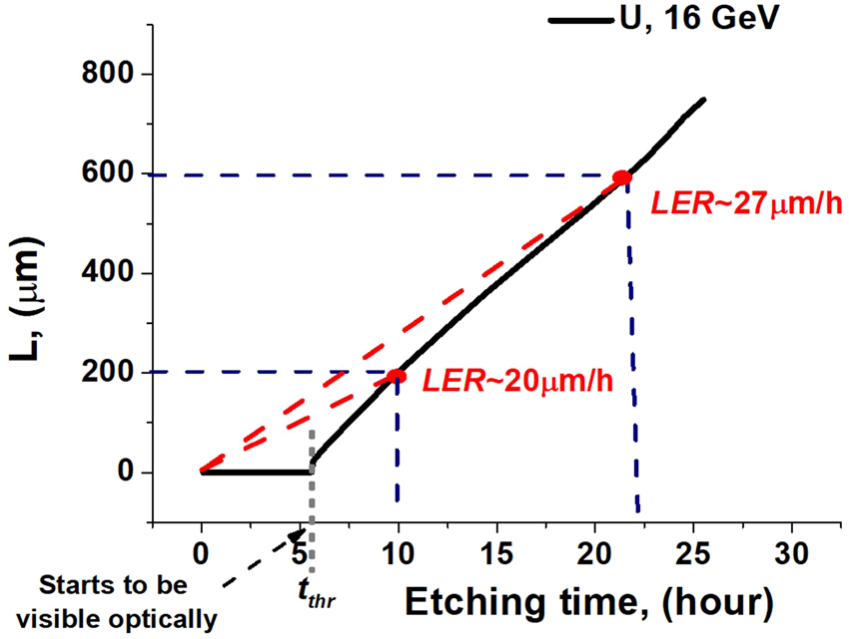
Simulated temporal dependence of the optically visible length of etched track of 16 GeV U ion in olivine.

Figure 14 demonstrates that at least 5 hours are necessary to overcome the 400 nm threshold of the optical visibility of the etched channel in a track of 16 GeV U ion. Since LER is defined as the visible etched length divided by the total etching time (${LER}={L}/t$), the registered LER is small at the initial times (or large *RR*). When the etching time increases the averaged LER also increases tending to its steady-state value at small *RR*.

## Effect of Diffusion Coefficients Variations in the Etched Track Core

Diffusion coefficients of reaction products and molecules of an etchant were assumed constant in the presented simulations. However, interactions with walls of a nanometric channel may decelerate moving of molecules decreasing these coefficients. Indeed, the authors of^35,36^ mentioned about variations of the diffusion coefficient of a liquid depending on the diameter of a nanometric channel. Also, molecular dynamics simulation of water diffusion in carbon nanotubes^37^ revealed the change of the diffusion coefficient by ~10 times when the diameter of a nanotube increases.

Dependence of the diffusion coefficient on the diameter of the nanometric etched channel may considerably affect etching kinetics of a track. For example, this effect may cause a difference in the optically detected etching kinetics of short ion tracks of the same lengths in olivine. This effect will be investigated in a separate paper.

## Conclusions

Traditional point of view on the track etching processes in minerals connects the LER with the SHI energy losses in a given segment of the trajectory^15^. This treatment is widely used in the literature (see e.g.^12,17^), However, it does not take into account peculiarities of the spatial distribution of the deposited energy around the ion trajectory. Our simulations demonstrated that information about the dependence of LER on the radial distributions of the deposited energy (or chemical activation) of a material along the whole projectile trajectory is very important for prediction and interpretation of etching results. This effect can be investigated in details by joint application of the presented numerical model and experimental methods which provide visibility threshold of etched channels comparable with the nanometric diameters of these distributions.

## Data Availability

All data generated and analyzed in this study are available upon request to the authors.
